# Lf2 is a knotted homeobox regulator that modulates leaflet number in soybean

**DOI:** 10.1111/tpj.70658

**Published:** 2026-01-12

**Authors:** Chancelor B. Clark, Denise Caldwell, Qiang Zhu, Jinbin Wang, Dominic Provancal, Austin C. Edwards, Qijian Song, Charles V. Quigley, Anjali S. Iyer‐Pascuzzi, Jianxin Ma

**Affiliations:** ^1^ Department of Agronomy Purdue University West Lafayette Indiana USA; ^2^ Center for Plant Biology Purdue University West Lafayette Indiana USA; ^3^ Department of Botany and Plant Pathology Purdue University West Lafayette Indiana USA; ^4^ Soybean Genomics & Improvement Laboratory USDA‐ARS Beltsville Maryland USA

**Keywords:** allelic variation, gene cloning, genetic mapping, leaflet development and morphology, leaflet number

## Abstract

Variation in leaf complexity modulates light capture and is a target for crop enhancement. Soybean (*Glycine max*) typically has compound leaves with three leaflets each, but a spontaneous mutation, designated *lf2*, possesses seven leaflets, offering a means to dissect the molecular mechanisms specifying leaflet number and assess its potential for soybean improvement. However, the developmental and genetic bases of the *lf2* mutation remain unknown. Here, we characterize the seven‐leaflet phenotype at morphological and developmental levels and identify the mutation responsible for the phenotypic changes. Microscopic examination of leaf emergence sites revealed that the seven‐leaflet phenotype arises through a two‐step process: five leaflets form initially, followed by secondary leaflet initiation at the margins of the central leaflet. Genetic mapping delineated *lf2* to an approximately 2.5 Mb region at the start of chromosome 11. Fortuitously, integration of pedigree analysis with comparative analysis of genomic sequences from the region pinpointed a 2‐bp deletion in the coding sequence of a gene, homologous to the Arabidopsis *KNAT7* encoding a KNOTTED1‐LIKE HOMEOBOX 2 transcription factor, as the sole candidate for *Lf2.* The deletion is predicted to result in disruption of the putative DNA‐binding homeodomain. Expression of the wild‐type allele of the candidate gene in the seven‐leaflet *lf2* mutant restored the three‐leaflet phenotype, validating its candidacy. Partial disruption of the wild‐type allele through CRISPR‐Cas9 editing induced extra leaflet formation. This study advances our understanding of leaflet formation and underscores the potential of targeted modifications at the *Lf2* locus as a strategy to achieve diverse soybean plant architecture.

## Significance Statement

This study identifies the genetic basis for a major soybean leaflet development mutant, *lf2* through an integrative genomics approach without fine mapping. The findings from the study lay a foundation for further dissection of the gene networks underlying this trait and facilitate the use of leaflet number variation in pursuit of optimized soybean shoot architecture.

## INTRODUCTION

Leaves, the primary organs responsible for capturing light for photosynthesis, arise on the fringes of shoot apical meristems and exhibit a wide range of morphological differences within and among species (Bar & Ori, 2014). Leaf development can be classified into three stages: initiation, during which leaf primordia emerge from the periphery of the shoot apical meristem; primary morphogenesis, where the marginal leaf structures and lamina are formed; and differentiation, when the leaf tissues expand and mature to their final form (Bar & Ori, 2015). Compound leaves are those in which the lamina is subdivided into leaflets, in contrast to simple leaves in which the lamina is a single undivided unit (Bharathan & Sinha, 2001). These separate leaflets arise during primary leaf morphogenesis through the formation of distinct leaflet primordia (Mo et al., 2022). Compound leaves have evolved independently many times within the plant kingdom. They may hold ecological advantages for rapid growth of pioneering plant species, help plants tolerate herbivory, or be adaptations for response to seasonal drought, although evidence for each of these ecological functions is limited (Malhado et al., 2010). In plant species, differences in leaf and leaflet structure play a major role in determining light interception, the basis for photosynthesis and ultimately yield (Falster & Westoby, 2003). As a result, modifications to leaf structures are a major target of crop improvement as part of the fine‐tuning of overall shoot architecture (Clark & Ma, 2023).

Compound leaf development results from the maintenance of the marginal blastozone (MB), a meristematic region capable of organogenesis (Blein et al., 2008). In many plant species, members of the *KNOTTED1‐LIKE HOMEOBOX I* (*KNOXI*) gene family play a central role in maintaining the MB. In some legumes, particularly in the inverted repeat‐lacking clade, this function is reported to be replaced in part by orthologs of *LEAFY* (*LFY*), which in Arabidopsis is associated with floral meristem identity (Bar & Ori, 2015). *KNOTTED1‐LIKE HOMEOBOX 2* (*KNOX2*) genes are also known to impact compound leaflet development, generally as negative regulators of leaf complexity that act antagonistically to *KNOX1* (Furumizu et al., 2015). Whereas *KNOXI* expression is closely associated with zones of undifferentiated cells, *KNOX2* genes are expressed in a broader array of differentiated tissues. In addition to compound leaf development, *KNOX2* genes have been implicated as important players in secondary cell wall development, stress response, and even nodule development (Di Giacomo et al., 2017; Iannelli et al., 2023; Wang et al., 2020).

Soybean, a critical crop for global oil and protein production, typically has two opposite simple (unifoliate) leaves at the first node after the cotyledons followed by single alternate compound leaves with three leaflets (trifoliate) at each subsequent node. Compound leaves are found in the majority of legume species, exhibiting diverse forms such as the soybean‐like trifoliate leaflet arrangements in common bean (*Phaseolus vulgaris*), mung bean (*Vigna radiata*), and cowpea (*Vigna unguiculata*), the four opposite paripinnate leaflets in peanut (*Arachis hypogaea*), and the many alternate imparipinnate leaflets in chickpea (*Cicer arietinum*) (Liu, Yang, et al., 2023). While the genetic basis of compound leaflet development is well characterized in model legume species, particularly *Medicago truncatula*, comparatively little is known about this trait in legume crops such as soybean.

Nevertheless, multiple studies have demonstrated that knocking down or knocking out *LFY* homologs in soybean results in reduced leaf complexity (Champagne et al., 2007; Wang et al., 2025). Additionally, genetic loci resulting in multifoliate (i.e., more than the typical three) leaflets have been reported (Fehr, 1972; Orf et al., 2006; Wang et al., 2001; Wang, Niu, et al., 2024). *Lf1* is an incompletely dominant mutation that confers a high rate of pentafoliate (five leaflets) leaves at the normally trifoliate nodes, which was mapped to a region with Glyma.08g281900, encoding an AP2‐domain transcription factor, as the candidate gene (Jeong et al., 2017). *lf2* is a recessive mutation that results in seven leaflets (heptafoliate) at the “trifoliate” nodes. *Lf1* and *lf2* display synergistic effects, with the *Lf1/Lf1;lf2/lf2* genotype having 12–14 leaflets per “trifoliate” node (Fehr, 1972). Despite the *lf2* allele first being reported more than five decades ago, the genetic mutation(s) responsible for the heptafoliate phenotype had not been identified. In this study, we report the development pattern of the *lf2* heptafoliate phenotype and cloning of the gene underlying this locus, as well as profiling of downstream targets of Lf2.

## RESULTS

### Alternation of spatiotemporal leaflet initiation is the developmental basis underlying the *lf2* mutant phenotype

**Figure 1:**
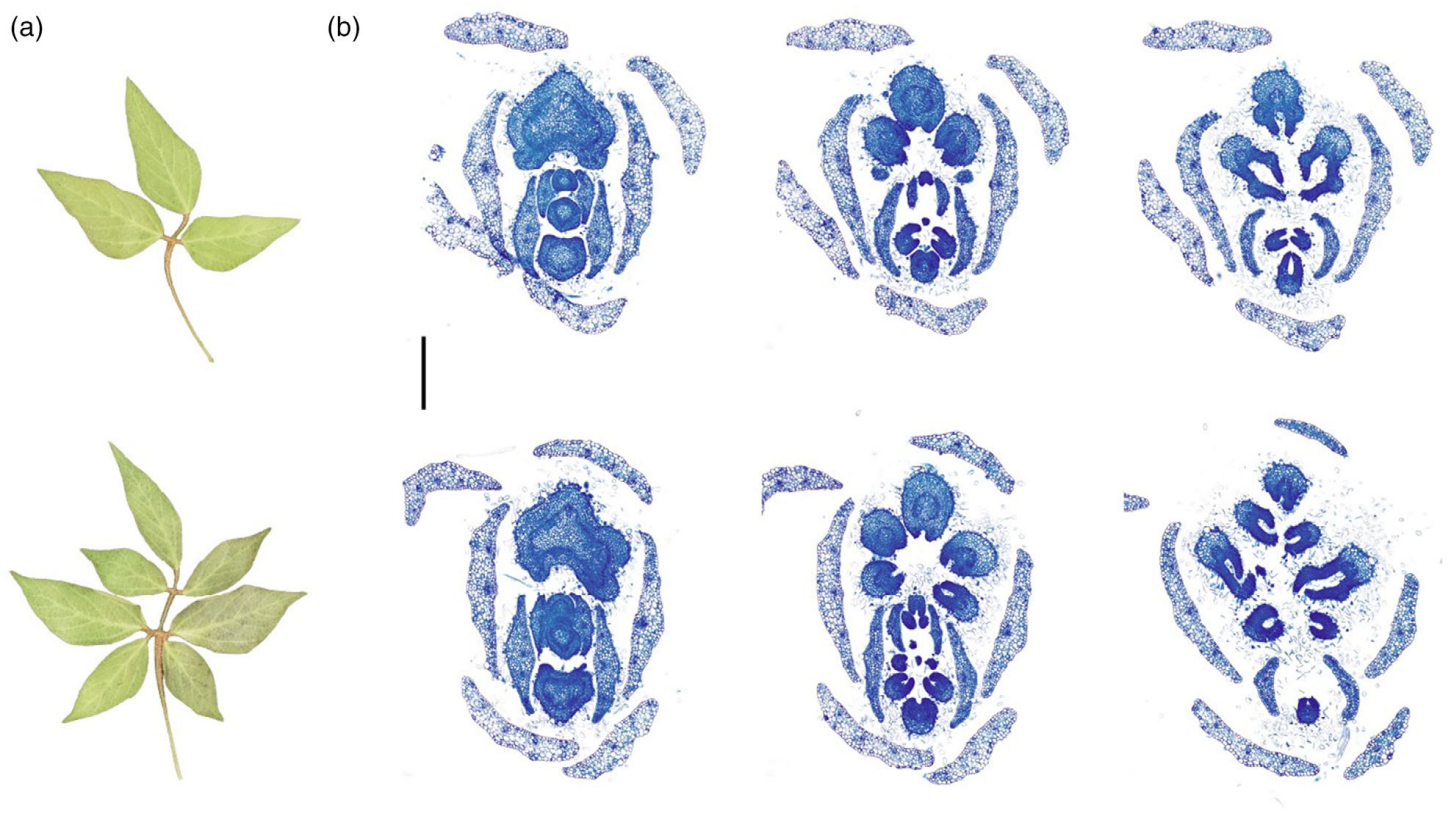
Characterization of *lf2* Mutant. (a) Mature leaves of Williams 82 (*Lf2/Lf2*, top, trifoliate) and L73‐1087 (*lf2/lf2*, bottom, heptafoliate). (b) Light microscope images of developing leaf primordia. Williams 82 (top) progresses from a single leaf primordium to a compound trifoliate leaf. L73‐1087 (bottom) progresses from a single leaf primordium to a palmately compound leaf with five leaflets. The central leaflet then undergoes secondary leaflet formation at the margins for a total of seven leaflets. The scale bar is 40 μm. Samples were collected from plants growing in the greenhouse.

To understand the *lf2* seven‐leaflet soybean trait, we evaluated the lines known to possess it. The seven‐leaflet trait was originally reported as a spontaneous mutation (i.e., the sudden appearance of an individual plant with seven leaflets) in the trifoliate cultivar Hawkeye (Fehr, 1972), and this line was designated as T255 (PI 548232). This trait was then backcrossed into the cultivar Clark (PI 548533) to develop the *lf2/lf2* Clark near‐isogenic line (NIL) L73‐1087 (PI 547580), one of many resources developed as part of the USDA soybean isoline collection (Gilbert et al., 2023). L73‐1087 has three leaflets on each of the opposite leaves at the first node (Figure S1), in contrast to the wild‐type unifoliate single leaflet, and seven leaflets on each subsequent node, in contrast to the wild‐type trifoliate (Figure 1a). The phenotype is consistent, although occasionally 6, 8, or 9 leaflets were observed instead of seven on individual nodes. Unlike other multifoliate soybean mutants, trifoliate leaves are never observed in L73‐1087 after the first node. In general, the total leaf area is greater in L73‐1087 than in typical wild‐type trifoliate soybeans (Fehr, 1972).

To trace the development of the seven‐leaflet phenotype, we performed light microscopy of cross‐sections of the meristem (Figure 1b). In trifoliate soybean leaflets, the terminal and two lateral leaflets emerge nearly simultaneously from the leaf primordium. In the heptafoliate mutant, initially five leaflets emerge in a palmately compound fashion, corresponding to the two lateral leaflets on each side and one central leaflet attached to the rachis. Subsequently, the central leaflet undergoes secondary leaflet formation as two lateral leaflets emerge from the leaflet margins. This secondary leaflet formation at the central leaflet follows a reiterative pattern that mimics the normal formation of trifoliate leaves in wild‐type soybean.

### Inheritance analysis of leaflet numbers suggests *Lf2* is incompletely dominant over *lf2*

To understand the inheritance pattern of the *lf2* mutation, we developed a mapping population by crossing the *lf2* mutant L73‐1087 to Williams 82 (*Lf2/Lf2*, trifoliate). The F_1_ hybrids had wild‐type unifoliate leaves at the first node and most subsequent nodes had wild‐type trifoliate leaflets, but each F_1_ plant produced an occasional node with four or five leaflets. In the F_2_ population, 89 individuals had seven leaflets per leaf, and 328 individuals had three or mostly three leaflets, consistent with a single gene modulating leaflet number in this population (*χ* ^2^ for 3:1 = 2.97, *P*‐value = 0.085). Trifoliate is incompletely dominant in this population, as evidenced by the occasional appearance of leaves with four, five, or six leaflets or extra lobes in the F_1_ and heterozygous F_2_ individuals.

### Bulked segregant analysis delimits *Lf2* to a terminal region on the long arm of chromosome 11

**Figure 2:**
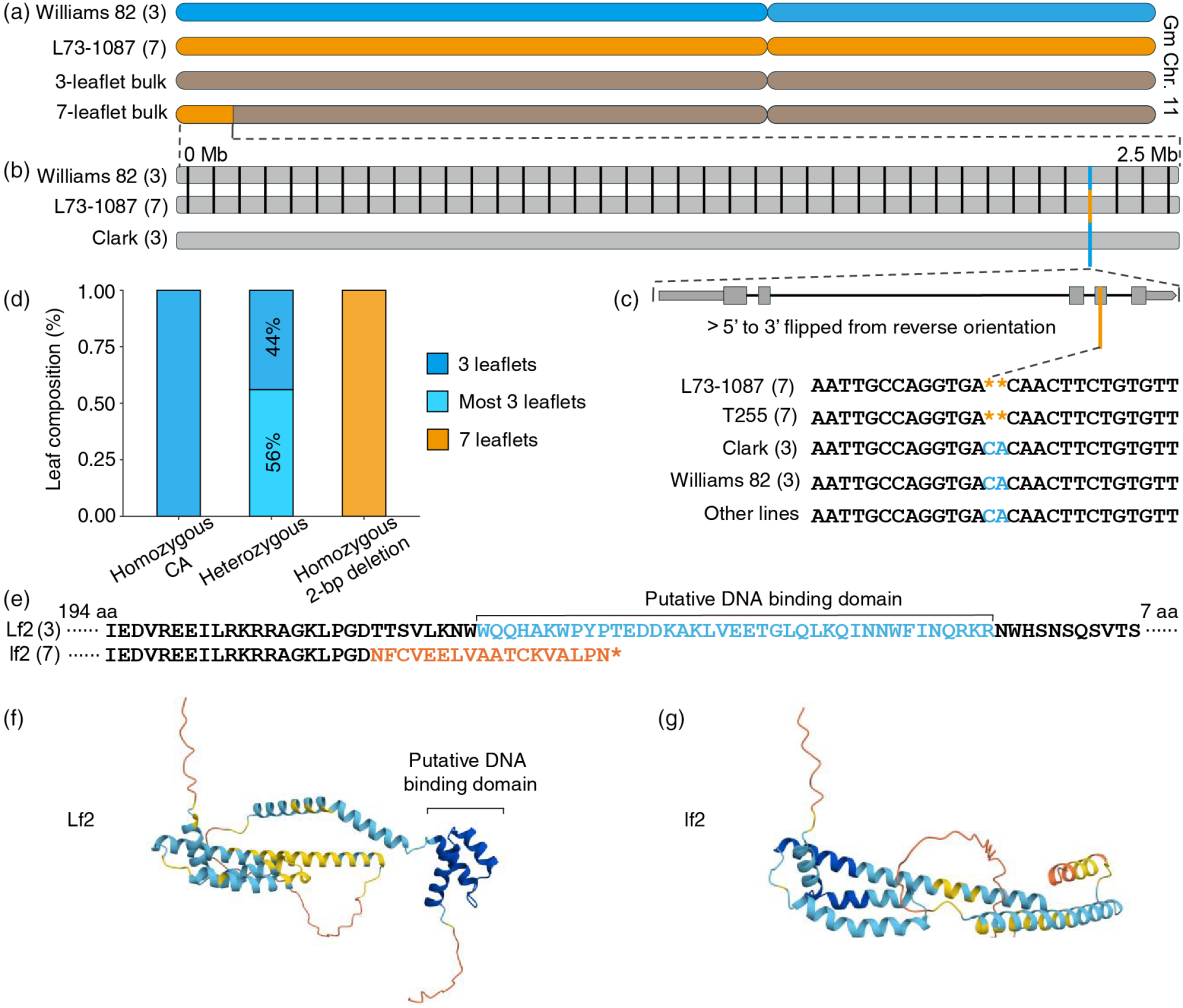
Identification of the genetic basis for *lf2*. (a) The location of the lf2 region on chromosome 11 as defined by BSA. The blue chromosome segments correspond to Williams 82, the orange to L73‐1087, and the blue‐orange stripes show heterozygous. (b) Genetic variation between Williams 82, L73‐1087, and Clark in the lf2 region defined by BSA. Each black bar represents a polymorphism between Williams 82 and L73‐1087 while the orange bar shows the only polymorphism between L73‐1087 and Clark. (c) The gene structure of Glyma.11g027100 and sequence alignment at the site of the 2‐bp deletion. The orange bar shows the location of the 2‐bp deletion in the fourth exon, the only mutation that can distinguish the 2 *lf2;lf2* seven‐leaflet lines from their *Lf2;Lf2* trifoliate counterparts. Note: The gene is on the reverse strand relative to the genome annotation but is flipped so that 5′ to 3′ is left to right for clarity. (d) Charts showing the phenotypic distribution for each allele at the 2‐bp deletion site demonstrating perfect correspondence between the genotype and phenotype. (e) The consequence of the 2‐bp deletion on the predicted protein sequence, eliminating the putative homeodomain DNA‐binding domain. The putative homeodomain is highlighted in light blue while the changed amino acids in the mutant are shown in orange. (f) Protein structure prediction for wild‐type Lf2 candidate with the putative DNA‐binding domain visible on the right side of the structure. (g) Protein structure prediction for the *lf2* mutant candidate.

To detect the genomic location of the gene responsible for the *lf2* seven‐leaflet phenotype, we carried out bulked segregant analysis (BSA) of typical trifoliate or heptafoliate individuals in the F_2_ population. BSA placed the *Lf2* locus at the beginning of chromosome 11 (Figure 2a). Markers ranging from 2 119 662 base pairs (bp) to 2 574 195 bp were homozygous for the L73‐1087 genotype in the heptafoliate bulked sample (Table S1). However, in the 50 k SNP marker dataset (Song et al., 2013) we used for genotyping, there were no markers displaying polymorphism between Williams 82 and L73‐1087 upstream of bp 2 111 286, so we considered the mapped region to be from 0 to 2 574 195 bp on chromosome 11 based on the principle of BSA. No other regions were homozygous in either bulked sample. *Lf2's* placement on chromosome 11 is consistent with older studies showing it belongs to classical linkage group 16 and molecular linkage group B1 (Devine, 2003; Seversike et al., 2008).

### Comparative genomics pinpointed a 2‐bp deletion within the 2.57‐Mb region, as the sole causal mutation for *lf2*

Since L73‐1087 is a near‐isogenic line developed by backcrossing the seven‐leaflet phenotype from a spontaneous seven‐leaflet mutant line observed in the cultivar Hawkeye (T255), into the recurrent trifoliate parent Clark, we expected to observe this introgression in the region defined by BSA on chromosome 11. Surprisingly, publicly available SNP genotyping data for L73‐1087, Clark, and T255 show introgressions of T255 alleles on chromosomes 2, 3, 15, and 17, but not on chromosome 11 (Table S2). This can be explained by the half‐sibling relationship between Hawkeye (Mukden × Richland) and Clark (Lincoln × Richland), which could both have inherited the region containing *Lf2* from Richland, masking the introgression (Figure S2). As a result of the low amount of genetic variation between Clark, Hawkeye, and Williams 82 in the region defined by BSA, we suspected that we could easily find only one or a small number of mutations which met the criteria of occurring in the heptafoliate lines L73‐1087 and T255 but not in the trifoliate lines from which they were derived, Clark and Hawkeye, nor in Williams 82.

To search for mutations that could distinguish the three‐ and seven‐leaflet lines, we carried out whole‐genome resequencing of L73‐1087. In the region defined by BSA, only a single mutation could distinguish the heptafoliate L73‐1087 from Clark, Hawkeye, and Williams 82, a 2‐bp deletion in the fourth exon of Glyma.11g027100 (Figure 2b,c). While there are 38 polymorphisms between Williams 82 and L73‐1087, only the 2‐bp deletion distinguishes L73‐1087 from Clark, Hawkeye, and Williams 82. We subsequently sequenced the Glyma.11g027100 locus in T255 and our F_2_ mapping population. As expected, the mutation is also present in T255, the original line in which the seven‐leaflet phenotype was reported (Figure 2c). In the F_2_ mapping population derived from L73‐1087 and Williams 82, there was a perfect correspondence between the mutation and the leaflet phenotype, with all lines homozygous for the deletion having seven leaflets and all lines homozygous for lacking the deletion having three leaflets (Figure 2d). Multifoliate (4 or more) leaflets were observed on at least one node in about half (56%) of heterozygous lines, consistent with a single gene model where trifoliate is incompletely dominant over heptafoliate.

Since the seven‐leaflet phenotype originated as a spontaneous mutation in the cultivar Hawkeye, and because the seven‐leaflet phenotype is not described in lines besides T255 and L73‐1087, we should not expect to find the 2‐bp deletion or other mutations in Glyma.11g027100 in publicly available soybean resequencing data. To confirm this, we checked ~3000 publicly available resequenced wild and domesticated soybean accessions utilizing the variome tool on the SoyOmics website (Liu, Zhang, et al., 2023). We find a complete absence of mutations altering the amino acid sequence of this gene in any line (Figure 2c). Together, these observations suggest Glyma.11g027100 as the candidate for *Lf2* and the 2‐bp deletion as the candidate causal mutation for the seven‐leaflet mutant phenotype.

Glyma.11g027100 is one of four soybean genes derived from two rounds of whole‐genome duplication events, which are predicted to encode Class II KNOTTED‐like homeobox (KNOX II) transcription factors (Schmutz et al., 2010). The 2‐bp deletion within the Glyma.11g027100 locus in the mutant is predicted to cause a frameshift for 18 amino acids and then a premature stop codon, shortening the annotated protein from 279 to 232 amino acids (Figure 2e), while the putative DNA‐binding homeodomain (from 223rd to 261st amino acids of the protein encoded by the wild‐type allele), which consists of three alpha helices as predicted by AlphaFold, is completely eliminated in the mutant (Figure 2f,g). These observations echo a previous study, which demonstrated that a transposon‐induced loss‐of‐function mutation within a KNOX II transcription factor gene, designated *MtKNOX4*, produces one or two extra leaflets on rachises compared with the trifoliate wild‐type (Wang et al., 2023). We found that Glyma.11g027100 is one of the four duplicates that are putative orthologs of *MtKNOX4* (Figure S3), further supporting the functional role of Glyma.11g027100 in regulating leaflet number in soybean. The paralog that diverged from Glyma.11g027100 in the most recent whole‐genome duplication event, Glyma.01g214800, retains a high level of similarity (97.13% amino acid identity) and shows comparable expression levels (Liu et al., 2020). Nevertheless, the 2‐bp mutation in one (i.e., Glyma.11g027100) of the four duplicates is fully responsible for the mutant phenotype, suggesting that the other three paralogs have functionally diverged from this *Lf2* candidate gene with respect to its role in regulating leaflet number.

### Complementation test validates the *Lf2* candidate and the causal mutation for seven‐leaflet phenotype

**Figure 3:**
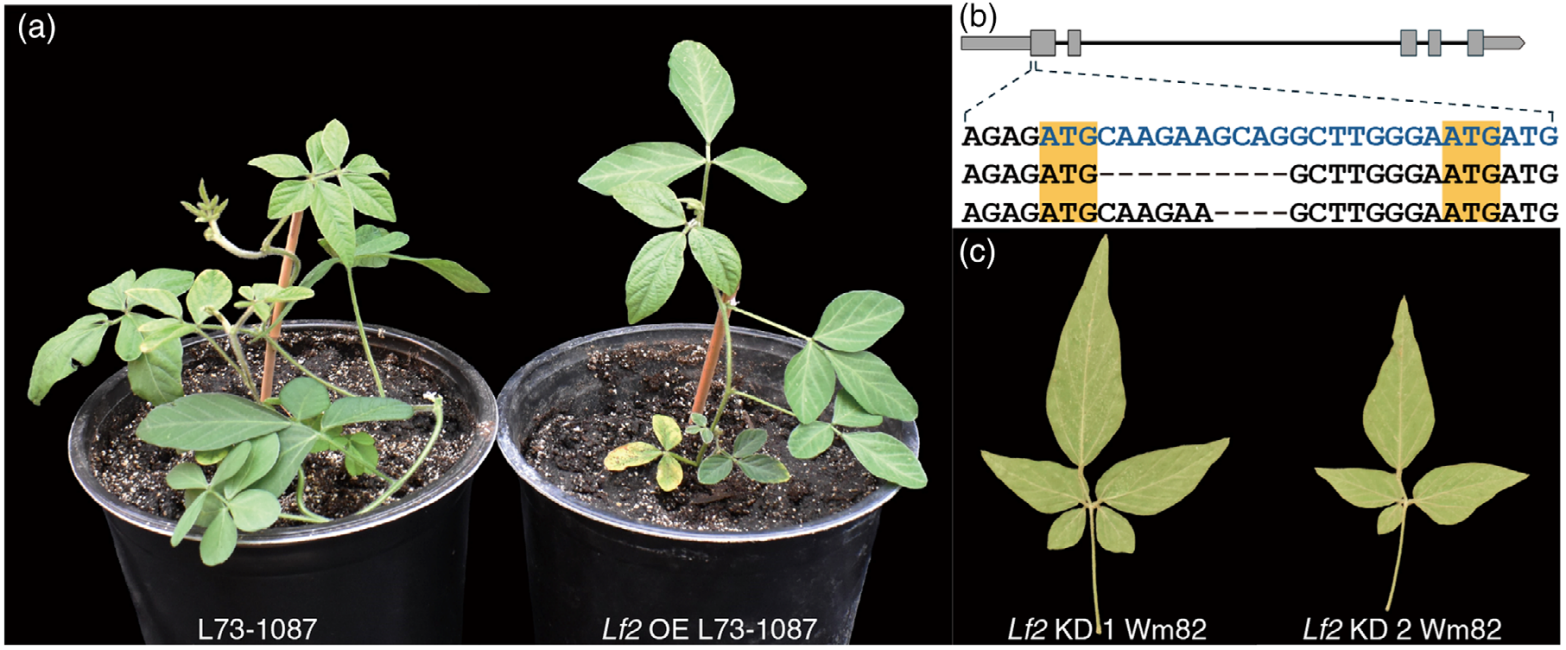
Validation of Glyma.11g027100 as *Lf2*. (a) Expressing the wild‐type Glyma.11g027100 CDS from Williams 82 under the influence of the CaMV 35S promoter in L73‐1087 restored the trifoliate phenotype. *Lf2* OE L73‐1087 shows a representative transgenic plant with the restored three‐leaflet phenotype. (b) The location within *Lf2* and sequence alignment of two CRISPR‐Cas9‐mediated editing events which disrupted *Lf2* in Williams 82. The top line of the alignment shows the beginning of the *Lf2* gene sequence in Williams 82, with the CDS in blue text. The second line of the alignment shows the 10‐bp deletion in the first event dubbed *Lf2* KD (knockdown) 1 Williams 82. The third line of the alignment shows the 4‐bp deletion in the second event dubbed *Lf2* KD 2 Williams 82. The orange highlights show the location of the canonical (first) and putative (second) alternative start codons. (c) Representative example leaves from the CRISPR/Cas9‐mediated editing events showing the additional leaflets.

To validate Glyma.11g027100 as the candidate for *Lf2*, we performed a complementation test by expressing the Williams 82 *Lf2* allele in the seven‐leaflet *lf2* line L73‐1087. Given that Glyma.11g027100 is expressed in various soybean tissues during plant development (Liu, Zhang, et al., 2023), the coding sequence of the *Lf2* allele was expressed in the transgenic lines under the control of the CaMV 35S promoter. We found that the transgene *Lf2* in the L73‐1087 background was able to rescue the three‐leaflet phenotype (Figure 3a). The transgenic lines from two independent transformation events had all or nearly all trifoliate leaves (one leaf with four leaflets was observed on one plant). While the complementation resulted in transition from heptafoliate to trifoliate, we did not observe further reductions in leaf complexity (e.g., simple leaves or leaves with less than three leaflets), clarifying the specific role of *Lf2* in maintaining leaflet number, as revealed by classical genetic analysis.

### *Lf2*‐knockdown lines created through CRISPR‐Cas9 exhibit extra leaflets

In theory, alteration of Lf2 through gene editing would result in increases in leaflet number, as a strategy to modify or optimize soybean plant architecture in any elite soybean varieties. To test this *de novo* approach to altering leaflet number, we designed guide RNAs (gRNAs) aiming to target all three highly unique editable sites (i.e., the only sites without identical or similar sequences in closely related genes) within the coding sequence of *Lf2* in Williams 82 (Figure S4). Among 25 independent transformation events, only two lines with variable edits at the first target site were obtained (Figure 3b). These two *Lf2*‐edited lines did not produce seven leaflets; instead, they exhibited high rates of four and five leaflets, as well as other leaf abnormalities such as extra lobes, including on the unifoliate leaves (Figure 3c; Figure S5). Event knockdown 1 (KD1) with a 10‐bp deletion displayed 40% of leaves with four or five leaflets, while event KD2 with a 4‐bp deletion displayed 15% of leaves with four leaflets. Gene function appears to have been partially retained in these lines, given that they do not show extra leaflets as consistently as the natural *lf2* mutant. As either of the two edits would make the original AUG an out‐of‐frame triplet (Figure S6), with inhibitory effect on translation initiation (Li et al., 2022), it is likely that similar to previous studies where a premature stop codon is induced near the beginning of a coding sequence (Sui et al., 2018; Wang, Zhai, et al., 2024), the in‐frame AUG near the edit sites in the mRNA is recognized as the start codon, enabling a slightly shortened open reading frame to encode a slightly truncated protein with the entire DNA‐binding homeodomain (Figure 3b; Figure S7).

### Subcellular localization of Lf2 and identification of its downstream targets through DAP‐seq

**Figure 4:**
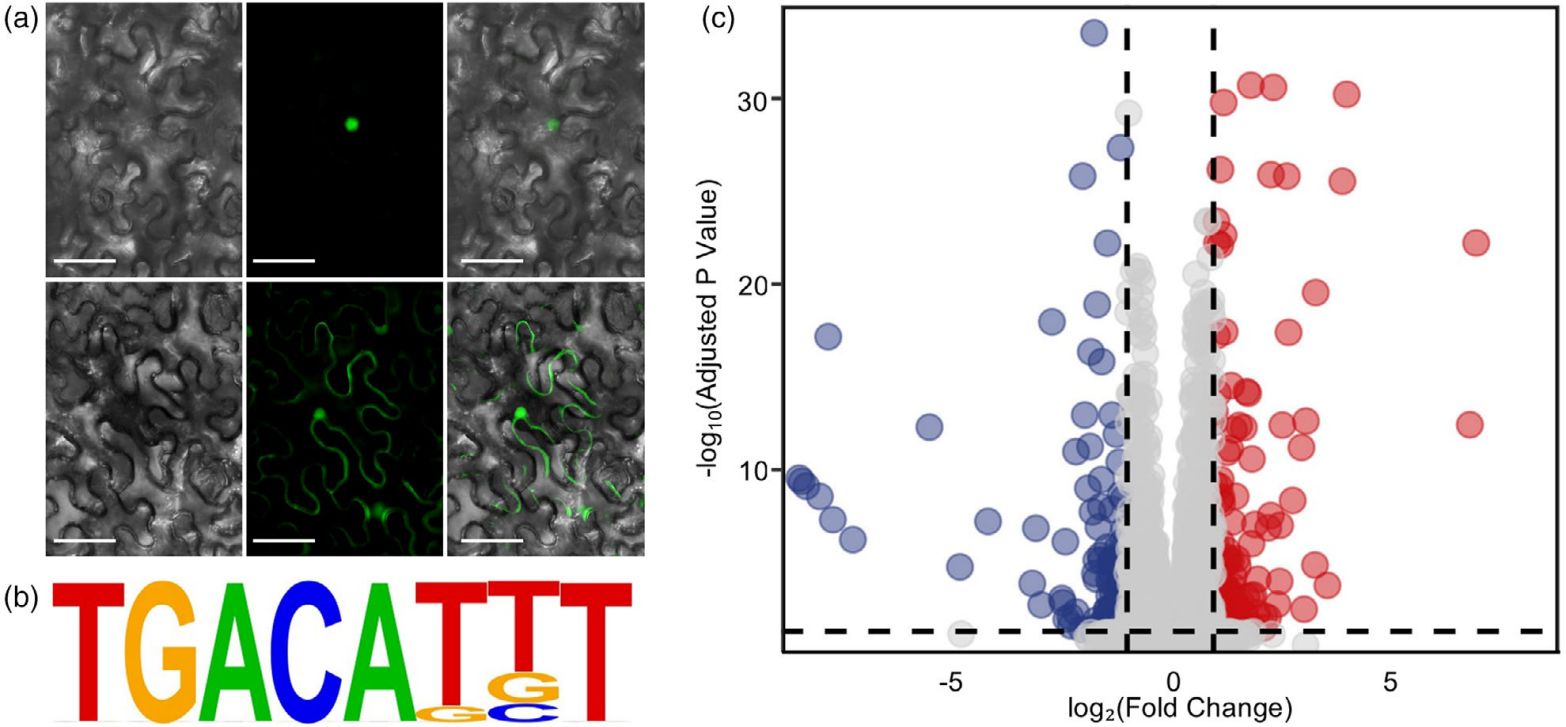
Regulatory profiling of *Lf2*. (a) Nuclear localization of *Lf2* in *Nicotiana tabacum*. Top from left to right shows Lf2 Brightfield, GFP, and Merged. Bottom row from left to right shows control GFP brightfield, GFP, and Merged. (b) The sequence logo corresponding to the putative binding site of *Lf2*, found in more than 60% of DAP‐seq peaks. (c) Volcano plot of total RNA sequencing results from Clark (*Lf2Lf2*) and L73‐1087 (*lf2lf2*). Genes significantly upregulated in the lf2 mutant are shown in red while genes significantly downregulated are shown in blue.

To validate the active role of Lf2 as a transcription factor, we performed subcellular localization of Lf2 fused with GFP fusion protein in tobacco. As expected, GFP signals were observed exclusively in the nuclei (Figure 4a). Having confirmed the nuclear localization of Lf2, we performed DNA Affinity Purification Sequencing (DAP‐seq), with two experimental replicates to identify the downstream targets of Lf2. A total of 344 putative Lf2‐binding sites were identified primarily in promoter regions of genes, including several with known or suspected functions in the specification of compound leaf development (Table S3). Analysis of the overrepresented motifs identified TGACAKBT as the most common shared sequence (Figure 4b; Figure S8), which is present in more than 60% of the Lf2‐binding sites detected by DAP‐seq and is consistent with homeobox transcription factor binding sites identified in other plant species (Ou et al., 2022). Notably, a specific site containing the motif TGACAGCT was found in the promoter region of Glyma.08G281900 (Figure S9), which was previously suggested as the candidate gene for *lf1* (Jeong et al., 2017). The detection of this site not only supports the genetically inferred epistasis between *Lf2* and *Lf1* but suggests their direct interaction regulates leaflet number, although the candidacy of Glyma.08G281900 as *lf1* remains to be functionally validated. Other Lf2 target genes detected by DAP‐seq potentially associated with leaflet development include Glyma.04G128800, a homolog of the *Arabidopsis thaliana* C2H2 zinc finger *SERRATE* (*SE*) (Prigge & Wagner, 2001), Glyma.14G120900 and Glyma.19G161000, homologs of the *A. thaliana* auxin efflux transporter *PIN6* and the AUX/IAA transcriptional regulator *ATAUX2‐11*, respectively.

### RNA sequencing of *Lf2* and *lf2* near‐isogenic lines identifies downstream effects of *Lf2*

To gain further insight into how the loss‐of‐function *lf2* allele changes patterns of gene expression, we performed total RNA sequencing (RNA‐seq) of vegetative 1 stage (when the first trifoliate leaf is fully emerged and unfolded) shoot apices of the Clark (*Lf2;Lf2*) and the Clark near‐isogenic line (NIL) L73‐1087 (*lf2;lf2*), with three biological replicates. A total of 246 genes were detected to be differentially regulated between Clark and the NIL, of which 130 were upregulated and 116 downregulated in L73‐1087 in comparison with Clark (Figure 4c; Table S4).

Among these were homologs of many genes known to be associated with compound leaflet development in other plant species. One gene significantly upregulated in the heptafoliate line is Glyma.01G198700, the most similar soybean gene to *M. truncatula PALM1*, which encodes a C2HC zinc finger superfamily protein well characterized for its essential role in compound leaf development (Chen et al., 2010; Ge et al., 2010). An *A. thaliana KNAT6* homolog, Glyma.18G141200, was downregulated in the heptafoliate line. Overexpression of the KNAT6 homolog *MtKNOX2* in *M. truncatula* results in increased leaflet number (Lu et al., 2024). Three homologs of *A. thaliana TCP1*, part of a key transcription factor family for compound leaf development in many species including tomato (Bi et al., 2024), were also found to be upregulated in the heptafoliate line. Two putative *BEL1* homeodomain genes, Glyma.02G060100 and Glyma.05G210300, were found to be differentially regulated. A BEL1 homolog in *M. truncatula*, MtPINNA1 (He et al., 2020), is a major player in maintaining trifoliate leaf development and BEL‐like, KNOX, and TCP transcription factors are reported to work together to regulate leaf margin development in *A. thaliana (*Yu et al., 2021). Additionally, the increased leaflet complexity observed in the tomato classical mutant *bipinnata* (*bip*) results from a mutation in a *BEL‐LIKE HOMEODOMAIN* (*BELL*) gene (Kimura et al., 2008; Nakayama et al., 2021). Glyma.08G281900, reported to be *Lf1*, is expressed at very low levels in both the Clark *Lf2* and Clark *lf2* NIL lines and shows no significant difference in expression. Clark possesses the *lf1* allele (rather than the functional *Lf1* allele), so we cannot rule out the possibility that a functional *Lf1* allele might be expressed at different levels in *Lf2* and *lf2* backgrounds.

## DISCUSSION

While not as ubiquitous among compound leaf development mutants as *KNOX 1* homeobox genes, which are positive regulators of leaf complexity across the plant kingdom, *KNOX 2* homeobox genes have been implicated in modulating this trait in several species. In fact, knocking out the putative ortholog of *Lf2* in the model legume species *M. truncatula*, *MtKNOX4*, also increased leaflet number, from the typical three to five in the mutant (Wang et al., 2023). This phenotype is similar to that caused by *lf2*, but distinct in several aspects. First, every leaf in the *lf2/lf2* mutant lines shows increased leaflet numbers while only around half of the *Mtknox4* leaves displayed increased leaflet numbers. Second, *Lf2* helps to regulate both primary and secondary leaflet formation in soybean, leading to seven leaflets rather than the five observed in the *Mtknox4* loss‐of‐function mutants. Third, *lf2* results in the typically unifoliate first node leaves becoming trifoliate, which was not reported by Wang et al. (2023) for *Mtknox4*. This could be reflective of divergences in gene function between soybean and *M. truncatula* or alternatively the observed phenotypic differences could be explained by nonequivalent loss‐of‐function alleles, where each mutation disrupts the gene in a slightly different way. Indeed, much remains unknown about compound leaflet development in soybean, providing many opportunities for deeper dissection of this trait.

Understanding the genetic basis for differences in leaflet number could prove useful for soybean improvement. A study of *Lf2/lf2* near‐isogenic lines showed that the seven‐leaflet phenotype had no effect or a negative effect on yield at higher planting densities (Seversike et al., 2009). However, this study also found that at low densities the seven‐leaflet phenotype could have advantages for yield due to increased light interception. This is consistent with seven leaflets being beneficial when light interception is a limiting factor and being a waste of resources once canopy closure is achieved. Rapid canopy closure and efficient light interception drive yield in many crop species (Sreekanta et al., 2024). Increased leaflet number on upper leaves likely contributes to increased shading of lower leaves, canceling out potential benefits from greater light interception. With the rapid development of gene editing technology such as CRISPR/Cas9 and methods for precise tracking of the spatiotemporal expression of genes, we are less constrained to only selecting between naturally occurring leaflet number phenotypes. We speculate that a putative soybean leaflet number ideotype of heptafoliate lower leaves and trifoliate upper leaves could strike the balance of increased light interception early in the growing season before canopy closure without causing increased shading or wasting resources in the upper canopy. This phenotype could be achieved through growth‐stage‐specific downregulation of *Lf2* or other genes in the leaflet development pathway. Having increased leaflet number only at the lower nodes of the plant would be analogous to reduced upper leaflet angle in maize, which facilitates the efficient capture of light at high planting densities (Mantilla‐Perez & Salas Fernandez, 2017).

Manipulation of soybean leaflet number, perhaps in combination with other soybean shoot architecture traits like branch angle (Clark et al., 2022; Virdi et al., 2023), could be a path to optimized soybean shoot architecture. Our targeted editing of *Lf2* at three sites through CRISPR‐Cas9 showed differential editing efficiencies, which may be relevant to multiple factors, such as target sequence and gRNA features, intrinsic properties, position effects, and differential mutation effects of the gene's function (Javaid & Choi, 2021). Alternative genome‐editing strategies may be explored to generate *Lf2*‐knockout lines for the seven‐leaflet phenotype as seen in the natural *lf2* mutant, or to engineer quantitative trait variation.

The main goal of this study was genetic analysis of the *lf2* mutant line to uncover the causal gene. Having identified *Lf2* as a *KNOX2* homeobox gene, we incorporated RNA‐seq and DAP‐seq as preliminary downstream analyses to place *Lf2* in the broader molecular context of leaflet development. The limited overlap between genes identified by DAP‐seq and those differentially expressed in near‐isogenic lines likely reflects the tissues sampled. These transcriptomic experiments were initiated before microscopy revealed that leaflet number is specified very early in primordium development. Moreover, the MB where leaflets are initiated comprises only a small fraction of the sampled shoot apices. As a result, bulk RNA‐seq may have limited power to capture the direct transcriptional regulation of *Lf2*. Future work using single‐cell RNA sequencing or microdissected tissues would be valuable in resolving this limitation. Nonetheless, the enrichment of homologs of known compound leaf patterning genes from across the plant kingdom indicates that our approach captured key downstream consequences of the *lf2* loss‐of‐function allele. Thus, while the central achievement of this study was identifying *Lf2*, these complementary assays provide a foundation for future exploration of this trait that can be used for crop improvement and understanding the broader trajectories of the genetic basis for leaflet number in legumes.

## MATERIALS AND METHODS

### Plant materials and mapping population development

The mapping population was developed by crossing Williams 82 to L73‐1087, using L73‐1087 as the pollen parent and Williams 82 as the female parent. About 15 F_1_ hybrid lines were confirmed by the appearance of purple flowers and black pods, dominant traits possessed by L73‐1087 but not by Williams 82. A total of 417 F_2_ individuals were grown in the field at the Purdue Agronomy Center for Research and Education (ACRE), in West Lafayette, Indiana, United States of America.

### Histology and light microscopy

Soybean meristems (2–5 mm) were excised from the region where the first true leaves emerged. The stem was cut just below the leaf emergence site and approximately 3 mm above it to isolate this zone. Larger leaves were removed by severing them at the petiole.

Samples were immediately fixed in a solution of 1.5% glutaraldehyde and 2% paraformaldehyde in 0.1 M sodium cacodylate buffer (pH 6.8) under low vacuum for 2 h, as previously described (Caldwell et al., 2017; Caldwell & Iyer‐Pascuzzi, 2019). Samples were washed in a buffer under vacuum and then subjected to a graded dehydration series using ethanol and tert‐butyl alcohol (TBA), with intermediate low‐vacuum treatments. Following three changes in 100% TBA over 24 h, tissues were infiltrated with molten paraffin at 54°C. Samples were embedded in molds, oriented perpendicularly, and solidified at room temperature.

Paraffin blocks were mounted to embedding rings, trimmed, and sectioned at 12 μm thickness using a rotary microtome. Sections were floated on the water at 34°C, mounted to slides, dried, deparaffinized, and stained with 0.05% toluidine blue using a graded xylene–ethanol series. Stained samples were mounted with Permount and coverglass and then imaged on an Olympus BX43 compound microscope equipped with a Spot Idea CMOS microscope camera.

### Phenotyping

Leaflet numbers were first phenotyped at the V2 stage when the first set of opposite “unifoliate” leaves and two sets of “trifoliate” leaves could be observed. These initial phenotypes were confirmed at the V5 stage.

### DNA isolation and bulked segregant analysis

F_2_ leaf tissue was collected at the V5 stage and DNA was isolated in 96‐well plates using a modified CTAB method (Mace et al., 2003). For BSA, two bulked samples were created. The first had leaves from 15 plants with seven leaflets and the second had 15 plants with three leaflets. Leaves in each of the two bulked samples were ground to a fine powder using liquid nitrogen. The ground bulked samples, as well as samples of Williams 82, L73‐1087, and Clark, were then isolated using the same CTAB method described above.

The five samples were then genotyped with the Illumina Infinium SoySNP50K BeadChip (Song et al., 2013). Each SNP genotype was classified manually using the GenomeStudio software from Illumina. The BSA mapping region was defined as the area in which the recessive (7 leaflet) bulk was homozygous for the recessive (L73‐1087) parent genotype according to the principle of BSA.

### Resequencing of L73‐1087

The same L73‐1087 DNA sample used for SNP genotyping was also used for resequencing. Sequencing was carried out by the Purdue Genomics Core (West Lafayette, IN, USA) and reads were sequenced to a depth of 20x coverage. Reads were aligned to the Williams 82 second annotation reference genome using the Burrows‐Wheeler Alignment tool (BWA) (Li & Durbin, 2009), and the data were processed using the SAMtools suite (Li et al., 2009). The region on chromosome 11 defined by BSA was compared with publicly available sequence data for Clark and Williams 82 manually using the Integrated Genome Viewer (IGV). Hawkeye's genotype was inferred using publicly available resequencing data for its parents, Richland and Mukden.

### Subcellular localization of Glyma.11g027100

Total RNA from Williams 82 young leaves was reversed transcribed using MMLV Reverse Transcriptase (Promega, Madison, WI, USA). Glyma.11g027100 CDS was amplified with Q5 High‐Fidelity Polymerase (New England Biolabs, Ipswitch, MA, USA). The CDS was then inserted into the vector pFM3100, under the influence of the Cauliflower mosaic virus 35S promoter and containing an N terminal green fluorescent protein (GFP) tag. This construct was transformed into the *Agrobacterium tumefaciens* strain EHA‐105. The *Agrobacterium* culture was diluted in magnesium chloride and injected into *Nicotiana tabacum* leaves. Agroinfiltration was conducted following the methods described in Wu et al. (2018). Two days after inoculation, transformed leaves were observed using an Olympus BX43 light microscope (Olympus Corporation, Hachioji, Tokyo, Japan) illuminated with an X‐Cite SERIES 120 Q Illumination System (Excelitas, Pittsburgh, PA, USA).

### Sanger sequencing of causal mutation

The following primers flanking the 2‐bp deletion were used to check the genotypes of T255 and the F_2_ mapping population. Forward: TCAAAACTTCACGTGCTAAACTC. Reverse: ATCTCTGCAGGGTTTCAAGTCA. PCR products were sent to Eurofins Genomics Sequencing Laboratory (Louisville, KY) for sanger sequencing using the forward primer listed above.

### Complementation test

Total RNA from Williams 82 young leaves was reverse transcribed using MMLV Reverse Transcriptase (Promega, Madison, WI, USA). Glyma.11g027100 CDS was amplified with Q5 High‐Fidelity Polymerase (New England Biolabs, Ipswitch, MA, USA) and inserted into the vector pPTN1171 (Ping et al., 2014) using the ClonExpress II One Step Cloning Kit (Vazyme, Nanjing, China). The following nested primers were used for amplification and insertion: outer F: ACCCTATATTATTCAACCCAATCCT, outer R: TGCTTGCTAGCTGTTCCACA, inner F: TTTACGAACGATAGCATGCAAGAAGCAGGCTTGGGA, inner R: TGATTTTTGCGGACTCTACCTCTTGCGTTTGGACT.

### Soybean transformation

The complementation construct was transformed into L73‐1087 via the *A. tumefaciens*‐mediated cotyledonary node method and regenerated through sterile soybean tissue culture as described in Wang, Duan, et al. (2024).

### DNA affinity purification sequencing (DAP‐seq)

Williams 82 genomic DNA (isolated as described above) and the pPTN1171 plasmid containing *Lf2* CDS were sent to CD‐Genomics (Shirley, NY, USA) who carried out DAP‐Seq Genomic Library Preparation, DAP‐Seq Protein Expression, and DAP‐seq‐binding assay and sequencing. Reads were aligned to the Williams 82 reference genome, second annotation first version (Wm82.a2.v1 assembly; Song et al., 2016), using BWA‐MEM (Li, 2013). Aligned reads were sorted, indexed, and filtered for uniqueness using SAMtools (Li et al., 2009). Two technical replicates were compared with the input genomic DNA and DAP‐Seq Binding Peaks (*q* value <0.05 and fold enrichment >2) were called using MACS2 (Feng et al., 2012). HOMER (Heinz et al., 2010) was used to find the most overrepresented sequences within the DAP‐seq peaks to identify potential binding motifs.

### RNA sequencing

RNA from 10 V0 Clark and L73‐1087 shoot apices for each of three replicates was isolated with Trizol Reagent (Invitrogen, Carlsbad, CA, USA). RNA quality and concentration were checked with Nano Drop (Thermo Fisher Scientific, Waltham, MA, USA). DNA was removed using the Turbo DNA Free Kit (Thermo Fisher Scientific, Waltham, MA, USA). Samples were sent to BMK Gene (Beijing, China) for total RNA sequencing.

Uniquely mapped reads were aligned to the Williams 82 (second annotation first version) reference genome using HISAT2 (Kim et al., 2019). Reads were formatted using SAMtools. Read counts were obtained using subread featureCounts (Liao et al., 2014). Differentially expressed genes were identified using DEseq2 (Love et al., 2014) and filtered to include only those with base mean above 10, adjusted *P*‐value (FDR) below 0.05 and an absolute log_2_ fold change ≥1.

## AUTHOR CONTRIBUTIONS

CBC and JM designed the research. CBC, DC, QZ, DP, ACE, QS, and CVQ carried out the research. CBC, JW, JM, DC, and ASI‐P analyzed the data. CBC wrote the manuscript with input from JM and the approval of all other authors.

## CONFLICT OF INTEREST

Authors declare no conflict of interest.

## Supporting information


**Figure S1.** The first node after the cotyledon in L73‐1087 (left) and Williams 82 (right) showing the normally simple unifoliate leaves becoming compound trifoliate leaves in the *lf2* mutant line.

**Figure S2.** Simple pedigree showing the relationship between L73‐1087, T255, Clark, and Hawkeye.

**Figure S3.** Phylogenetic tree of *Lf2* and closely related genes in soybean, *Arabidopsis*, and *Medicago truncatula*. Glyma.11g027100 (*Lf2*) and its closest match in *M. truncatula*, Medtr5g011070 are bolded.

**Figure S4.** Gene structure of Glyma.11g027100 showing the location of the CRISPR‐Cas9 target sites.

**Figure S5.** CRISPR‐Cas9 edited *Lf2*‐knockout lines showing examples of four and five leaflets.

**Figure S6.** Putative amino acid sequences using the alternative in‐frame ATG start codon.

**Figure S7.** Protein structure comparison (a) wild‐type Lf2 (b) putative edited KD1 Lf2.

**Figure S8.** Top 10 overrepresented motifs from HOMER analysis of Lf2 DAP‐seq peaks.

**Figure S9.** DAP‐Seq peaks in the *Lf1* promoter.

**Table S1.** SNP genotypes of bulked samples and parents in the region defined by BSA.

**Table S2.** SNP Markers in the BSA Region from publicly available resequencing data showing that the introgression of T255 DNA cannot be seen due to the close relationship among Clark and Hawkeye.

**Table S3.** Genes with Lf2 DAP‐seq peaks in their gene bodies or promoters.

**Table S4.** Differentially expressed genes between the Clark *Lf2Lf2* and *lf2lf2* near‐isogenic lines.

## Data Availability

The transcriptomic, genomic, and DAP‐Seq data were deposited in the NCBI Sequence Read Archive (Bioproject: PRJNA1271583) with the following BioSample: SAMN48877703 (L73‐1087 Resequencing), SAMN48899944 (Lf2 DAP‐Seq Rep1), SAMN48899945 (Lf2 DAP‐Seq Rep2), SAMN48899946 (Input Control for DAP‐Seq), SAMN48897410 (Clark Shoot Tip RNA Seq Rep 1), SAMN48897411 (Clark Shoot Tip RNA Seq Rep 2), SAMN48897412 (Clark Shoot Tip RNA Seq Rep 3), SAMN48897413 (L73‐1087 Shoot Tip RNA Seq Rep 1), SAMN48897414 (L73‐1087 Shoot Tip RNA Seq Rep 2), SAMN48897415 (L73‐1087 Shoot Tip RNA Seq Rep 3). The data can be accessed at https://www.ncbi.nlm.nih.gov/sra/?term=PRJNA1271583.
