# Supplementary figures and images for: Lf2 is a knotted homeobox regulator that modulates leaflet number in soybean
Source: Plant J. 2026 Jan 12;125(1):e70658. doi: 10.1111/tpj.70658 (PMC12795305; doi:10.1111/tpj.70658)

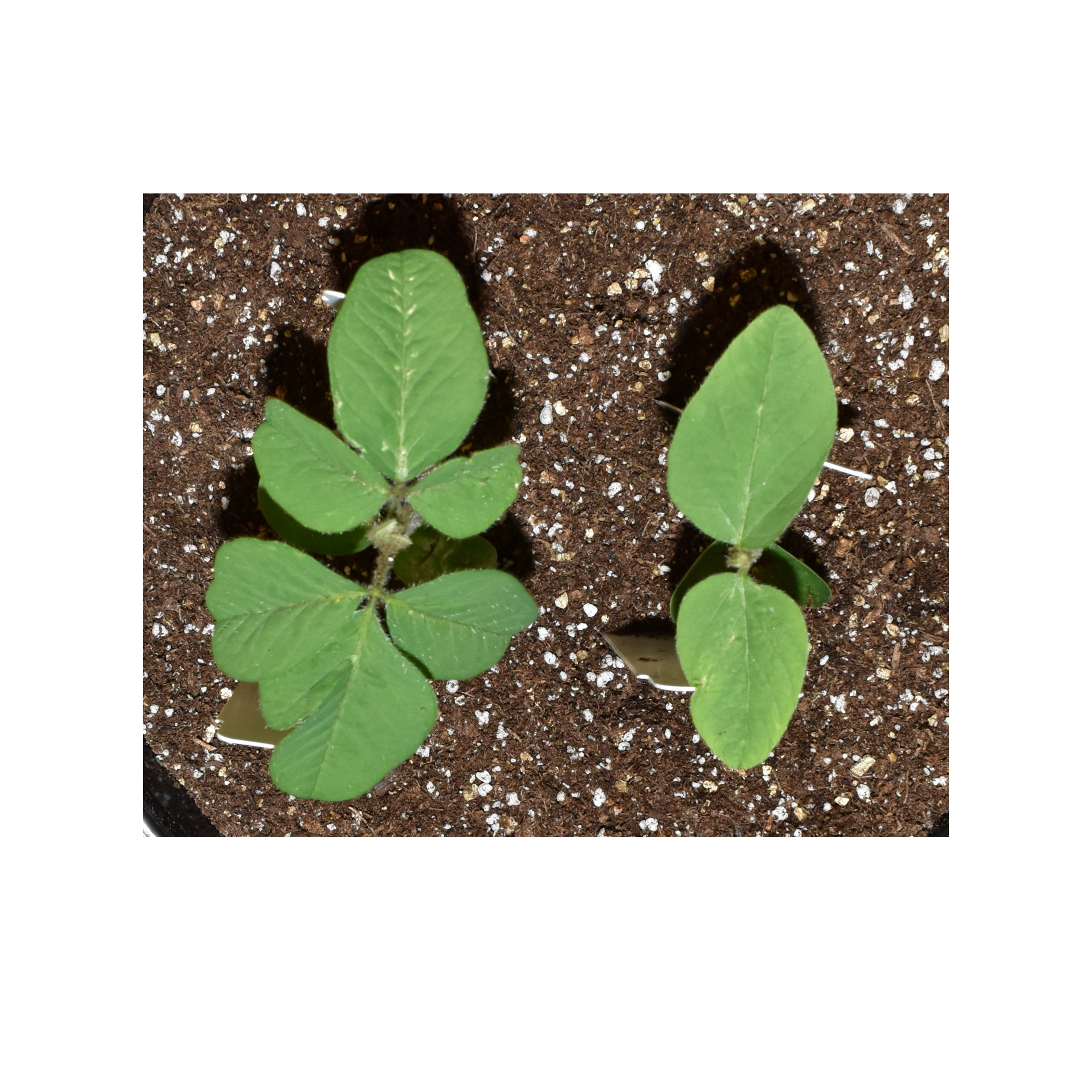

Supplement: Supplementary file 1 — Figure S1. The first node after the cotyledon in L73‐1087 (left) and Williams 82 (right) showing the normally simple unifoliate leaves becoming compound trifoliate leaves in the lf2 mutant line. [file TPJ-125-0-s008.png]

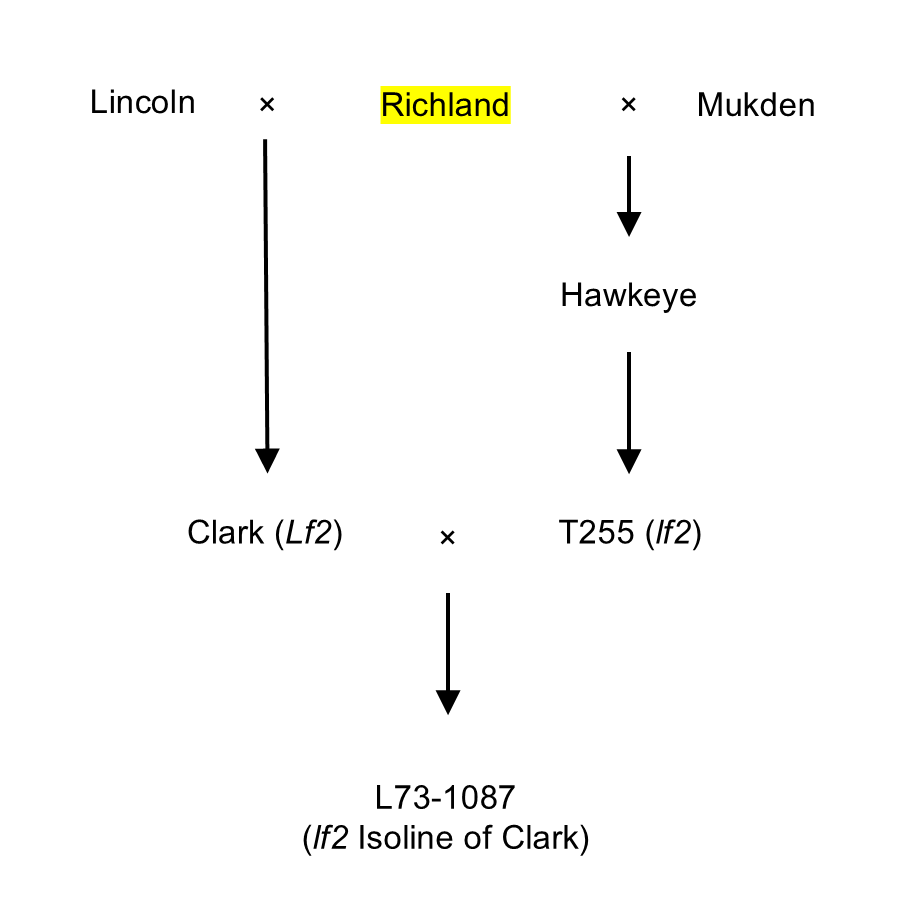

Supplement: Supplementary file 2 — Figure S2. Simple pedigree showing the relationship between L73‐1087, T255, Clark, and Hawkeye. [file TPJ-125-0-s005.png]

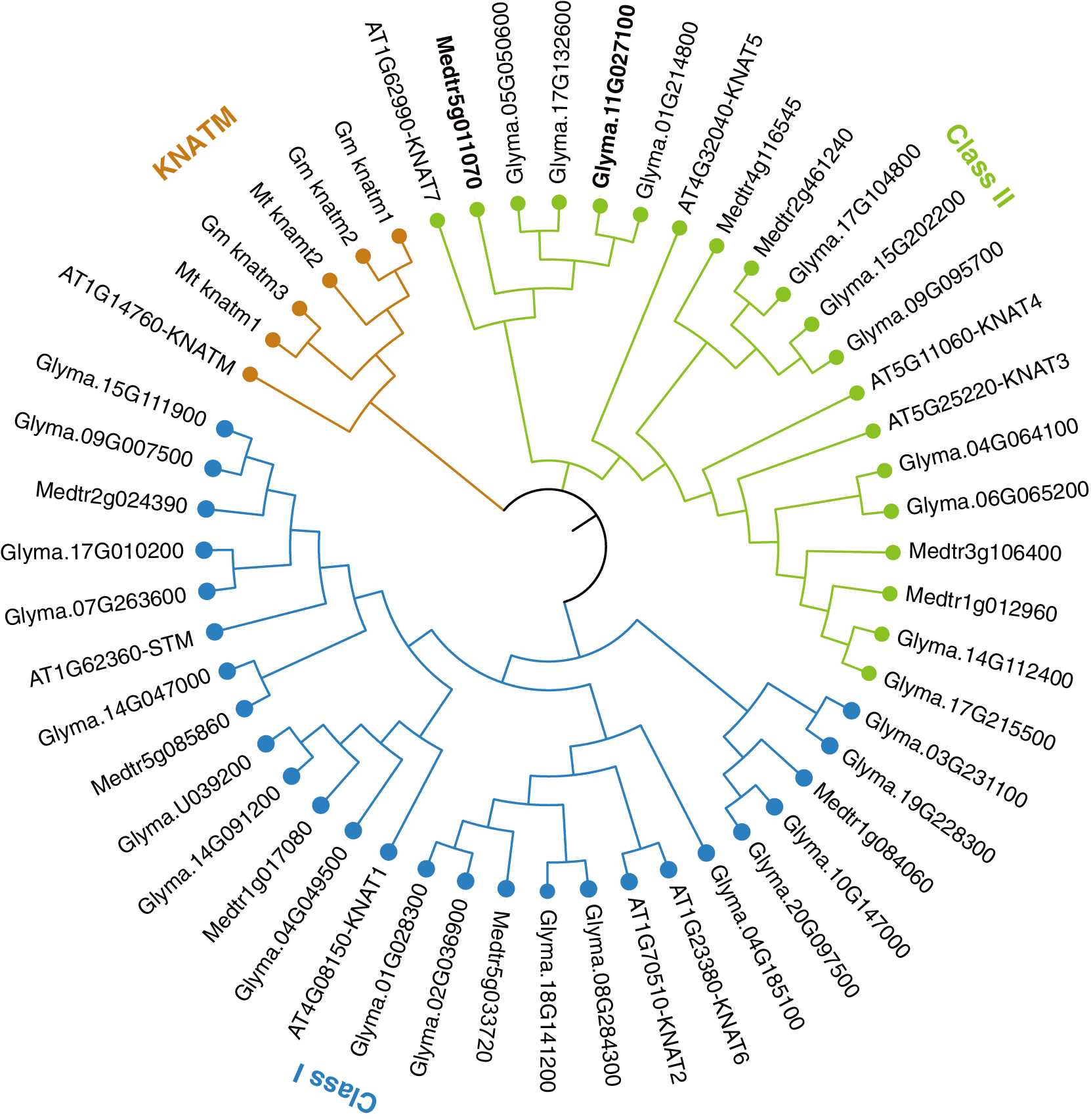

Supplement: Supplementary file 3 — Figure S3. Phylogenetic tree of Lf2 and closely related genes in soybean, Arabidopsis, and Medicago truncatula. Glyma.11g027100 (Lf2) and its closest match in M. truncatula, Medtr5g011070 are bolded. [file TPJ-125-0-s002.png]

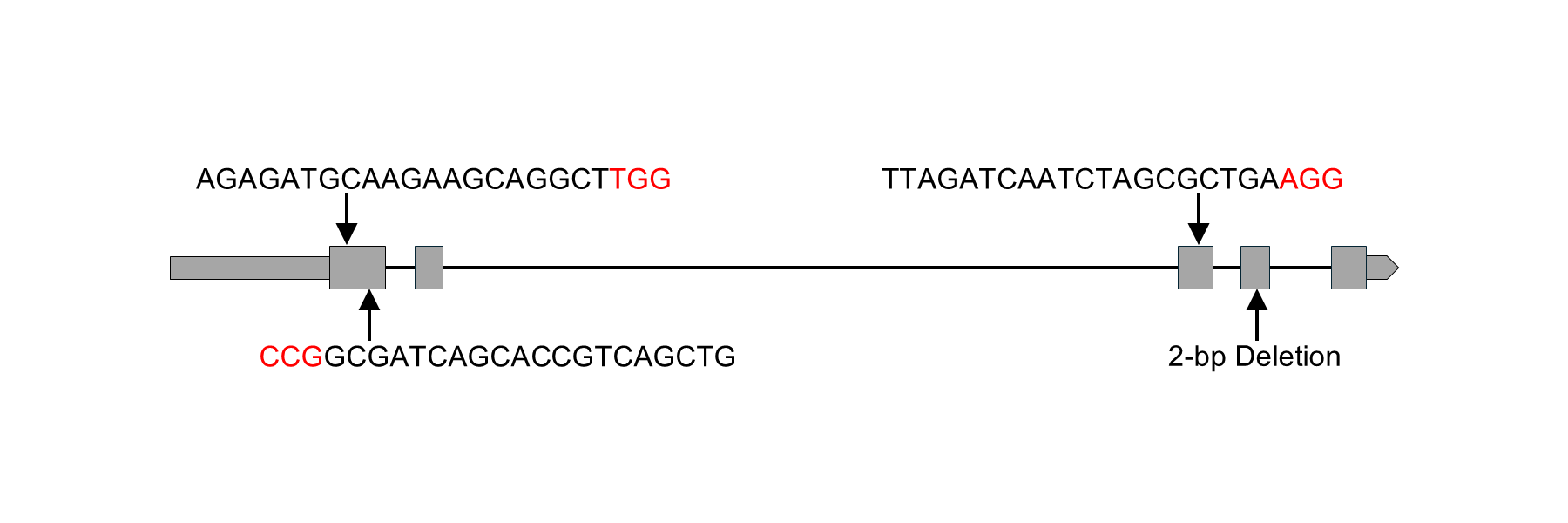

Supplement: Supplementary file 4 — Figure S4. Gene structure of Glyma.11g027100 showing the location of the CRISPR‐Cas9 target sites. [file TPJ-125-0-s012.png]

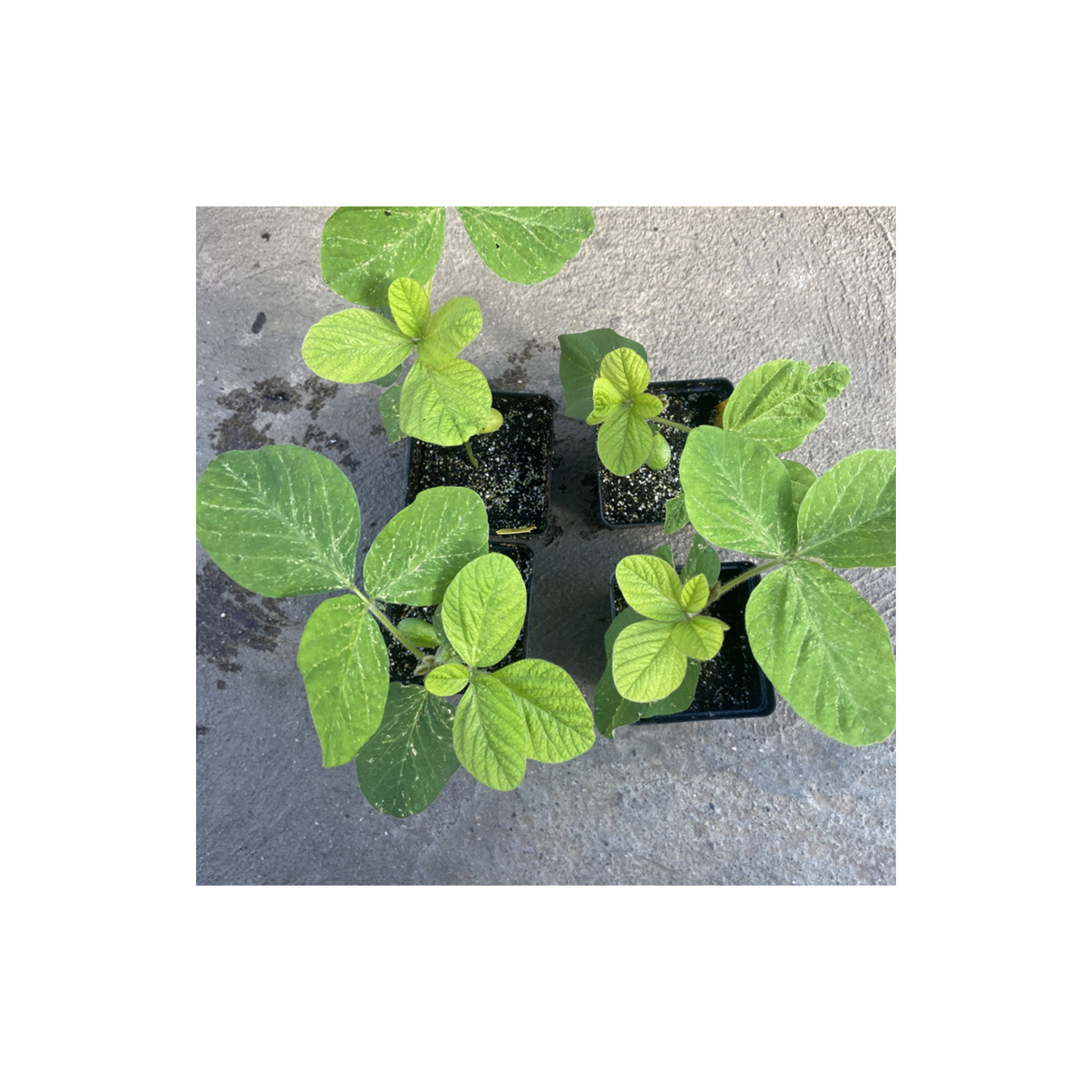

Supplement: Supplementary file 5 — Figure S5. CRISPR‐Cas9 edited Lf2‐knockout lines showing examples of four and five leaflets. [file TPJ-125-0-s003.png]

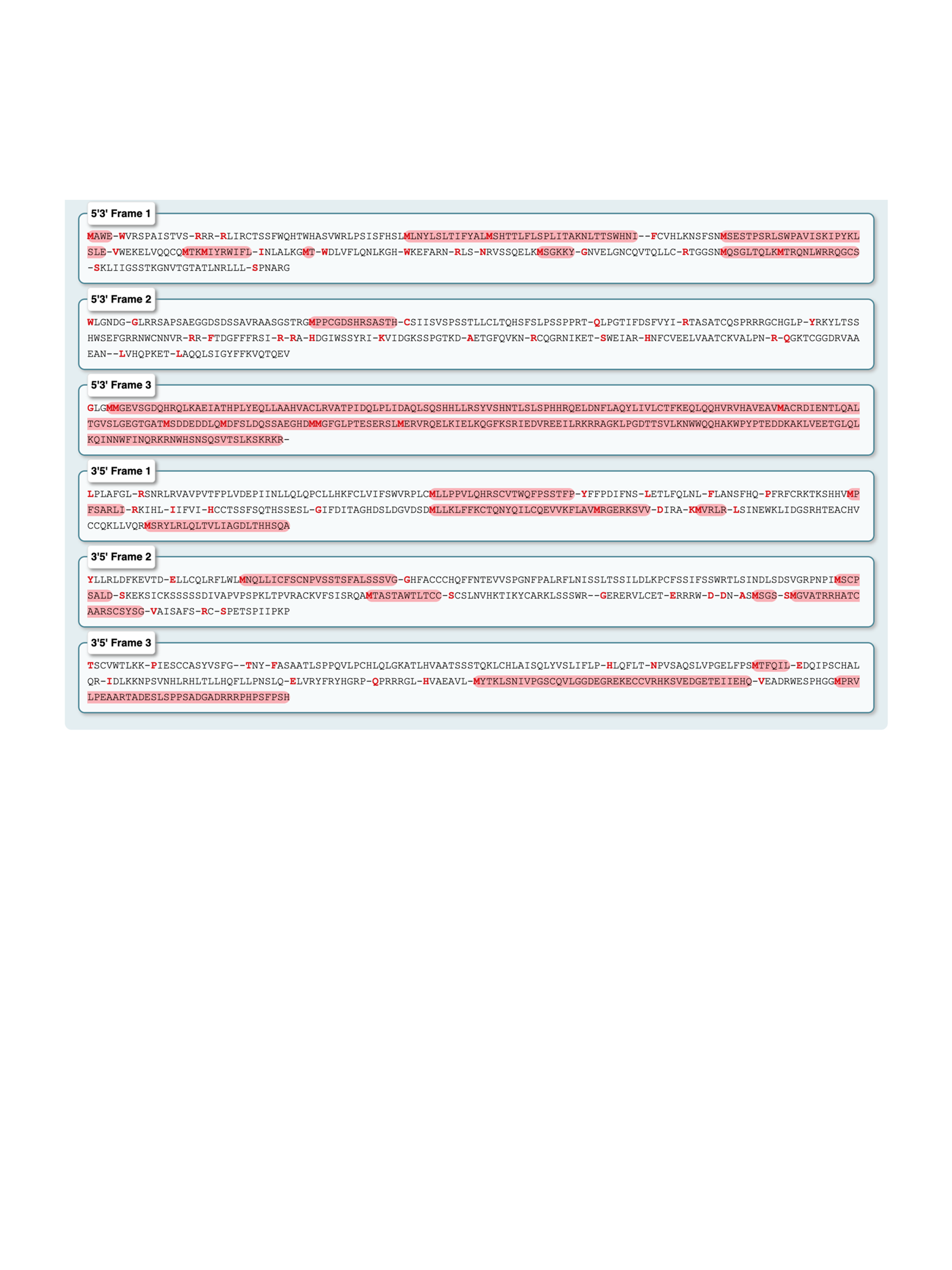

Supplement: Supplementary file 6 — Figure S6. Putative amino acid sequences using the alternative in‐frame ATG start codon. [file TPJ-125-0-s004.png]

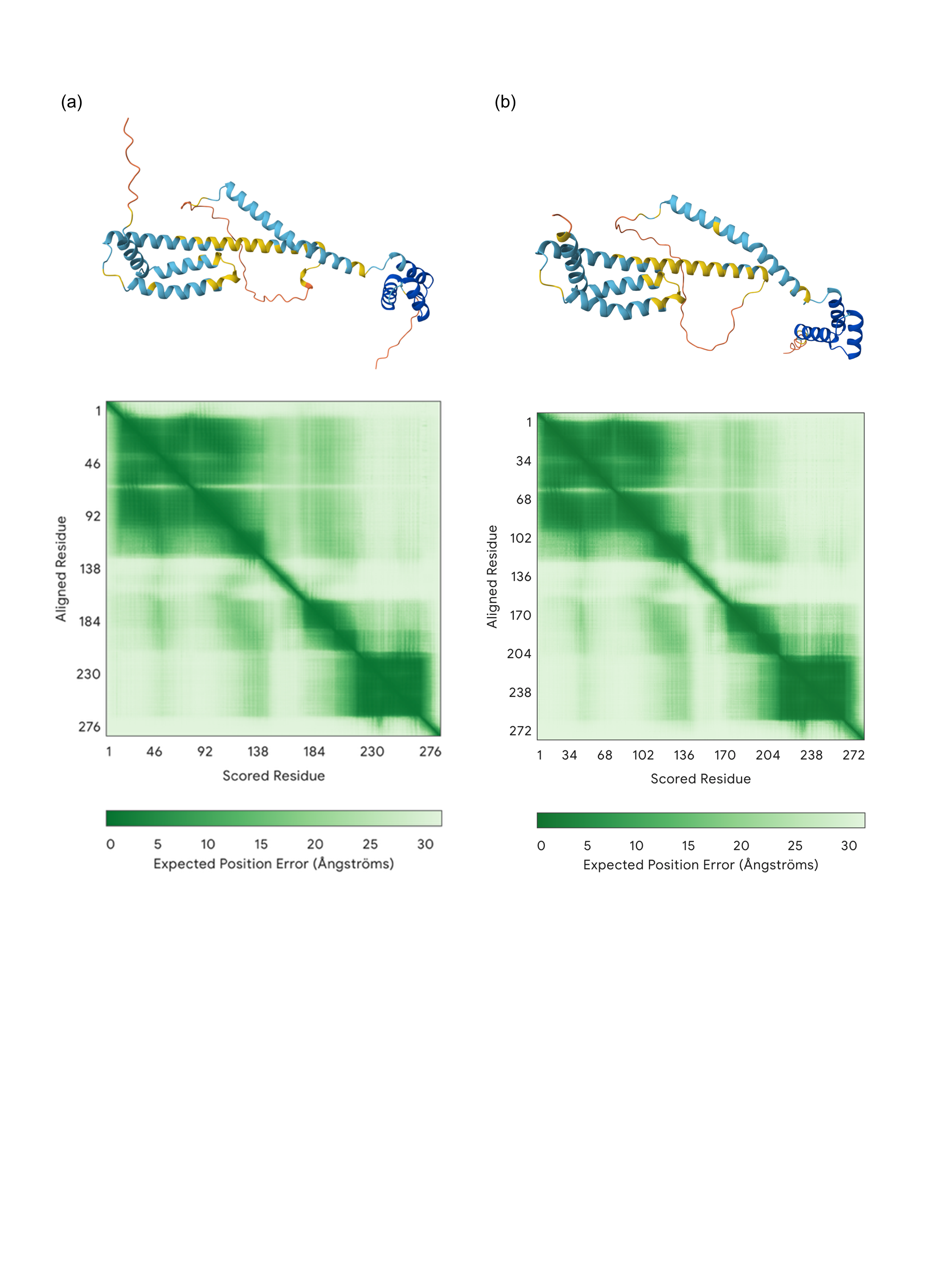

Supplement: Supplementary file 7 — Figure S7. Protein structure comparison (a) wild‐type Lf2 (b) putative edited KD1 Lf2. [file TPJ-125-0-s011.png]

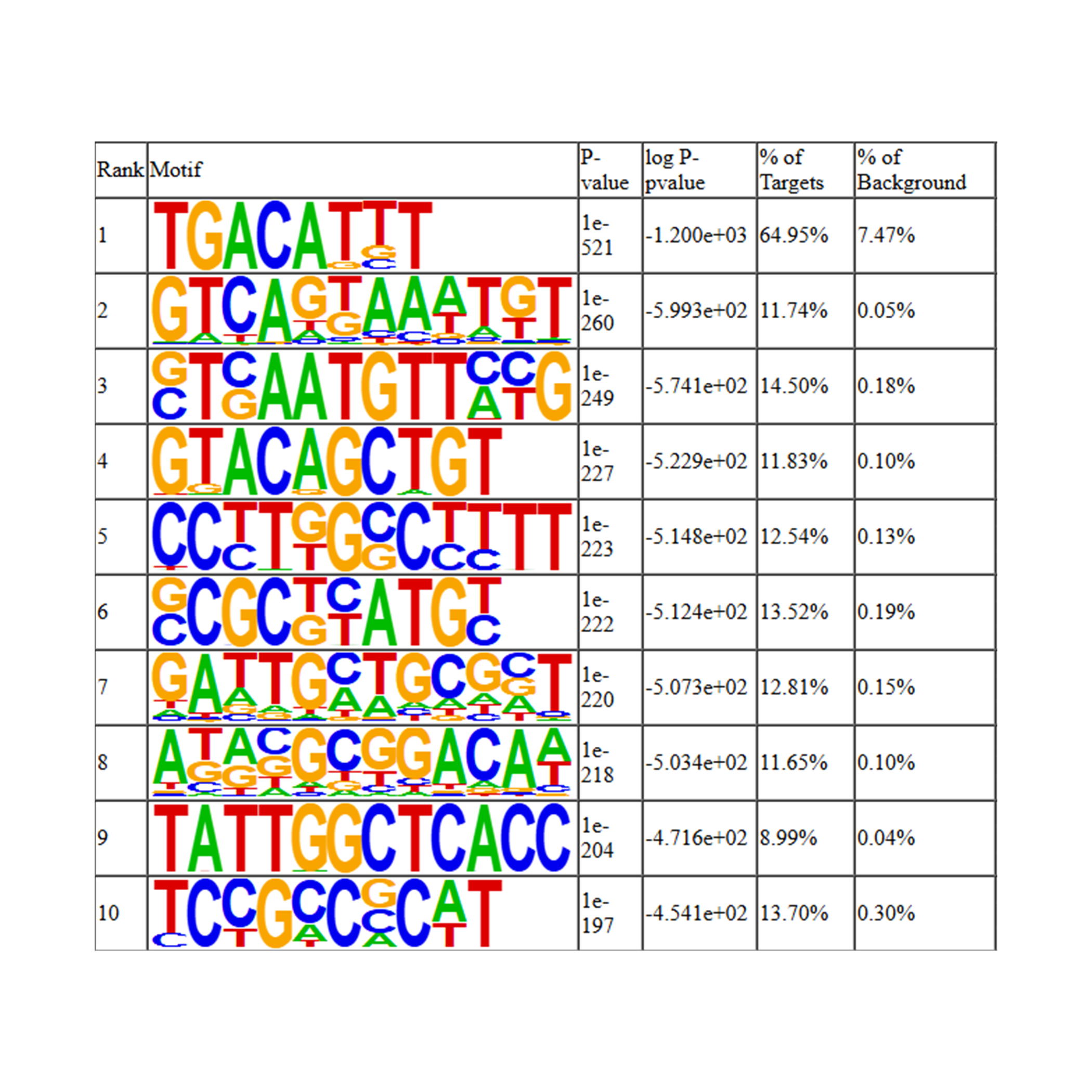

Supplement: Supplementary file 8 — Figure S8. Top 10 overrepresented motifs from HOMER analysis of Lf2 DAP‐seq peaks. [file TPJ-125-0-s001.png]

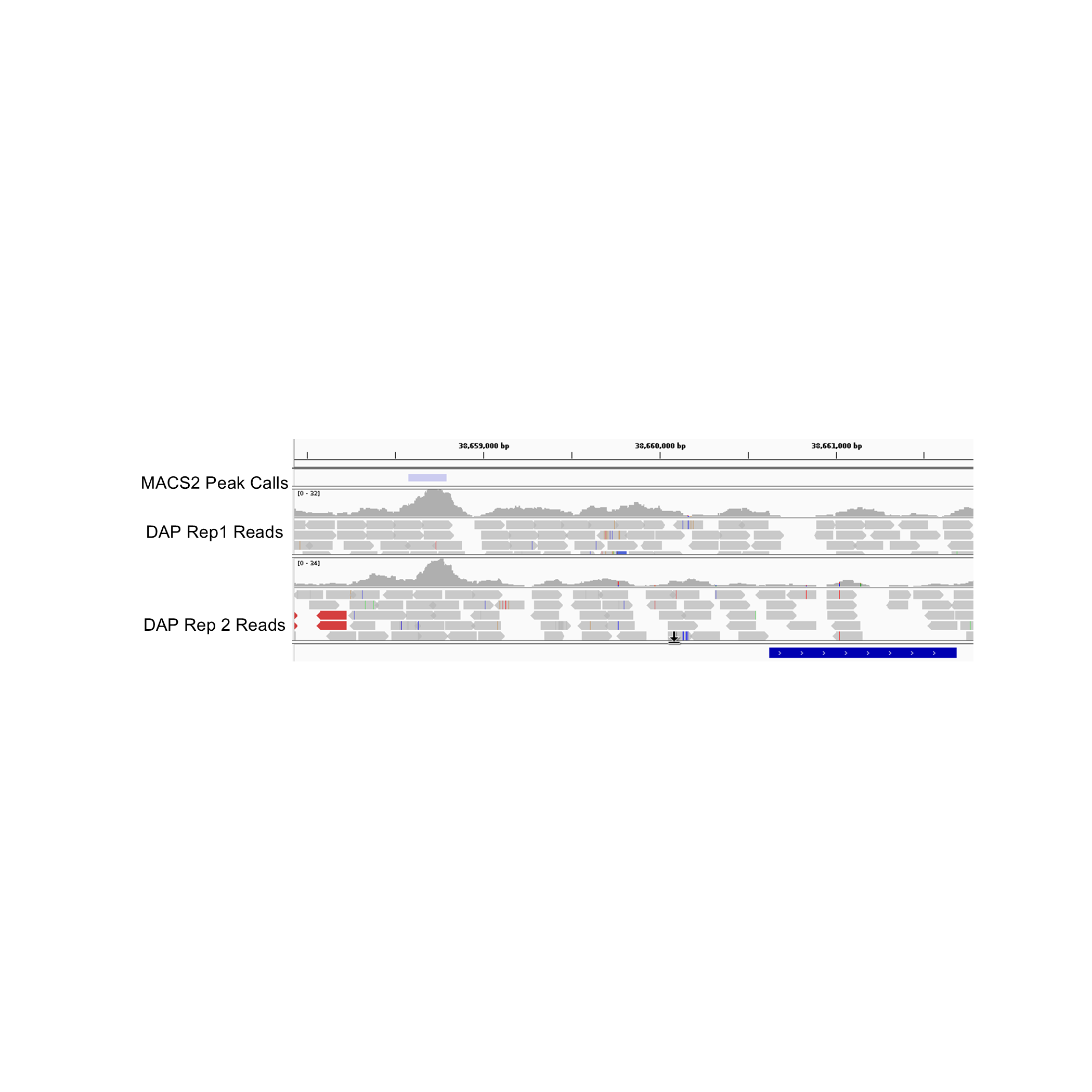

Supplement: Supplementary file 9 — Figure S9. DAP‐Seq peaks in the Lf1 promoter. [file TPJ-125-0-s009.png]
